# Burden of asthma exacerbations and health care utilization in pediatric patients with asthma in the US and England

**DOI:** 10.1002/iid3.299

**Published:** 2020-03-28

**Authors:** Mugdha Gokhale, Takako Hattori, Lee Evitt, Warren Lenney, Beth Nordstrom, Jenna Collins, Anna Schultze, Melissa K. Van Dyke

**Affiliations:** ^1^ Epidemiology, GlaxoSmithKline Upper Providence Pennsylvania; ^2^ Global Respiratory Franchise, GlaxoSmithKline London UK; ^3^ Value Evidence and Outcomes, GlaxoSmithKline London UK; ^4^ Global Medical Expert, GlaxoSmithKline London UK; ^5^ Respiratory Child Health Keele University Staffordshire UK; ^6^ Data Analytics, Evidera Waltham Massachusetts; ^7^ Real World Evidence, Evidera London UK

**Keywords:** asthma, health care, pediatrics

## Abstract

**Background:**

Data on asthma burden in pediatric patients are limited; this real‐world study investigated exacerbation frequency and health care resource utilization (HCRU) in pediatric asthma patients from the US and England.

**Methods:**

Data from pediatric patients (aged 6‐17 years) in the Optum claims database (US) or Clinical Practice Research Datalink with linkage to Hospital Episode Statistics (England) were analyzed. Patients were categorized into four hierarchical groups: treated asthma (patients with ≥1 baseline asthma medication), severe asthma (plus Global Initiative for Asthma Step 4/5), severe refractory asthma ([SRA] plus ≥2 baseline severe asthma exacerbations), and eosinophilic SRA (SRA plus blood eosinophil count ≥150 cells/µL). Exacerbation frequency and HCRU during the 12 months postindex were described.

**Results:**

Of 151 549 treated asthma patients in the US, 18 086 had severe asthma, 2099 SRA, and 109 eosinophilic SRA. There were 32 893 treated asthma patients in England, of whom 2711 had severe asthma, 265 SRA, and 8 eosinophilic SRA. In the 12 months postindex, ≥1 exacerbation occurred in 12.4% and 10.8% of patients with severe asthma, and 32.6% and 42.6% with SRA in the US and England, respectively. The proportions of patients with ≥1 asthma hospitalization in the 30 days after the first asthma exacerbation were 2.7% and 4.4% (treated), 3.5% and 8.2% (severe asthma), and 6.0% and 16.8% (SRA) in the US and England, respectively.

**Conclusion:**

This study provides insights into current asthma management practices in the US and England and indicates that some patients with severe disease have an unmet need for effective management.

## Graphical abstract

Real‐world data from the US and England were analyzed to investigate exacerbation frequency and health care resource utilization (HCRU) in pediatric asthma patients. Exacerbation frequency and HCRU tended to increase with asthma severity, indicating that disease burden increases with disease severity. This study provides insights into current asthma management practices in the US and England and indicates that some patients with severe disease have an unmet need for effective management.

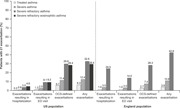

## 1 INTRODUCTION

Asthma in children and adolescents poses a substantial burden not only on global health care systems but also on the lives of the patients and their families.^1^, ^2^ Around 5% of pediatric patients with asthma have severe disease,^3^, ^4^ which is characterized by uncontrolled symptoms despite treatment with high‐dose inhaled corticosteroids (ICS) and/or oral corticosteroids (OCS).^5^, ^6^ Severe asthma accounts for over 50% of asthma‐related costs and is associated with more frequent exacerbations (or asthma attacks) than nonsevere asthma.^7^, ^8^, ^9^, ^10^ Furthermore, severe asthma or uncontrolled asthma is associated with increased health care resource utilization (HCRU), with patients more likely to require OCS treatment and emergency department (ED) visits or hospitalization.^11^, ^12^, ^13^, ^14^, ^15^

The prevalence of asthma in both children and adolescents is increasing globally.^1^ However, reported prevalence estimates vary greatly between countries,^16^, ^17^, ^18^, ^19^ likely owing to differences in country socioeconomic statuses, environmental factors, and patient access to treatment. The use of different disease definitions also makes it challenging to compare data between countries.^20^ Understanding the real‐world burden of disease and HCRU in pediatric patients with asthma would likely help to ensure resources to optimize disease management are directed to those patients with unmet needs. Furthermore, the existing limited information on the global burden of pediatric asthma is difficult to compare across regions given the differences in health care settings and treatment conventions between countries, as well as the differing methodologies used to define study populations. With this in mind, this study characterized patients aged 6 to 17 years with asthma in terms of exacerbation frequency, treatments prescribed and HCRU, using real‐world data from the US and England. Similar data analysis methodology was used across both datasets and data from the same time period were analyzed.

## 2 METHODS

### 2.1 Study design

We report results from two retrospective cohort studies (GlaxoSmithKline IDs: 208944; PRJ3070) using data from two databases: the Optum claims database (US population) and the Clinical Practice Research Datalink (CPRD) General Practice Online Data database with linkage to Hospital Episode Statistics data (England population). In each study, the population comprised pediatric patients who had ≥1 asthma diagnostic code recorded between 1 January 2011 and 31 December 2015. The index date was defined as the date of the most recent asthma record when the patient met all other inclusion and exclusion criteria. The England study was approved by the Independent Scientific Advisory Committee of CPRD. The US study used fully deidentified retrospective claims data, and as such, was not classified as research involving human participants as defined by 45 CFR 46.102(f)(2). Therefore, institutional review board approval and informed consent were not required.

### 2.2 Study population

In addition to ≥1 asthma diagnostic code (Table S1) during the cohort definition period, eligible patients were 6 to 17 years of age at index, had been prescribed ≥1 asthma medication during the 3 months before index, and had been continuously enrolled/registered in the respective databases during the 12‐month period before and including the index date (baseline period) and the 12 months after the index date (follow‐up period). Any patient with a diagnosis of cystic fibrosis (both populations) or with a code denoting “asthma resolved” during the 12‐month baseline or follow‐up periods (England population only) were excluded. Patients in the CPRD database were also required to be eligible for the Hospital Episode Statistics linkage.

Patients were classified into asthma severity categories based on the Global Initiative for Asthma (GINA) guidelines (GINA Steps 1‐5).^7^ Four hierarchical asthma groups were used, reflecting increasing asthma severity: (a) the treated asthma group (patients with ≥1 baseline asthma medication), (b) the severe asthma group (previous group restricted to patients meeting the GINA Step 4/5 criteria), (c) the severe refractory asthma group (previous group restricted to ≥2 baseline severe asthma exacerbations), and (d) the severe refractory eosinophilic asthma group (previous group restricted to baseline blood eosinophil count ≥150 cells/µL).

### 2.3 Outcomes and assessments

Patient demographics, exacerbations, clinical characteristics, and GINA step classification during the baseline period were recorded. If available, blood eosinophil counts nearest to the index date were recorded within a period of 6 months preindex to 6 months postindex. Outcomes recorded in the follow‐up period included the frequency of severe asthma exacerbations, and all‐cause and asthma‐specific HCRU (office visits, outpatient visits [US population only], ED visits, hospitalizations, prescription medications dispensed) recorded during the 30 days following the first severe asthma exacerbation. Among patients who had ≥1 asthma‐related hospitalization discharge during the follow‐up period, the frequency of hospital readmission within 30 and 90 days following the first observed hospital discharge during the follow‐up period was determined. The number of asthma‐related medications prescribed within 30 days following the first‐hospital discharge during the follow‐up period was also determined.

Severe asthma exacerbations were measured as any type of exacerbation or separately as exacerbations resulting in hospitalization, ED visit, or a short course of high‐dose OCS. Exacerbations occurring within 7 days of a previous event were considered to be part of the same exacerbation.

### 2.4 Statistical analysis

Descriptive statistics were performed separately for each of the four asthma groups for each country; no comparative analyses were conducted.

## 3 RESULTS

### 3.1 Patient population

**Figure 1:**
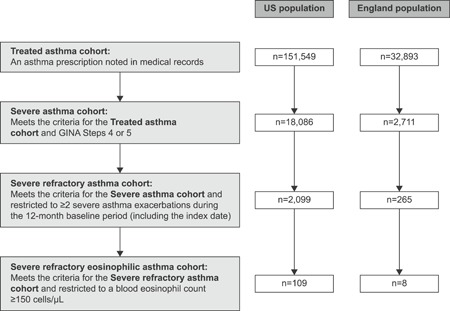
Overview of the hierarchical asthma severity groups in this study. The total numbers of patients who had an asthma diagnosis within the cohort definition period and were of any age in the US and England populations were 2275, 845, and 517 369, respectively. GINA, Global Initiative for Asthma

Overall, there were 151 549 and 32 893 pediatric patients (6‐17 years of age) included in the treated asthma group, 18 086 (11.9%) and 2711 (8.2%) patients included in the severe asthma group, 2099 (1.4%) and 265 (0.8%) patients included in the severe refractory asthma group, and 109 (0.1%) and 8 (0.02%) patients included in the severe refractory eosinophilic asthma group in the US and England, respectively (Figure 1). The severe refractory eosinophilic asthma group from England was not analyzed further owing to very small patient numbers. Demographic and clinical characteristics by the group are shown in Table 1. The median (interquartile range) age at index across all groups ranged from 10.0 (8.0‐14.0) to 12.0 (9.0‐15.0) years in the US and 12.0 (9.0‐15.0) to 14.0 (11.0‐16.0) years in England. Across all groups, the proportion of patients experiencing ≥1 exacerbation during the baseline period ranged from 24.5% to 100.0% in the US and from 12.4% to 100.0% in England.

**Table 1:** Demographic and clinical characteristics

	US population				England population^a^		
	Treated	Severe	Severe refractory	Severe refractory eosinophilic	Treated	Severe	Severe refractory
Total, n	151 549	18 086	2099	109	32 893	2711	265
Sex, male, n (%)	87 230 (57.6)	10 770 (59.5)	1262 (60.1)	68 (62.4)	18 909 (57.5)	1454 (53.6)	130 (49.1)
Age at index date, y, median (IQR)	11.0 (8.0, 14.0)	12.0 (9.0, 15.0)	10.0 (8.0, 14.0)	11.0 (9.0, 16.0)	12.0 (9.0, 15.0)	14.0 (11.0, 16.0)	13.0 (10.0, 16.0)
Age group, y, n (%)							
6‐11	77 721 (51.3)	8990 (49.7)	1261 (60.1)	56 (51.4)	14 282 (43.4)	819 (30.2)	104 (39.2)
12‐17	73 828 (48.7)	9096 (50.3)	838 (39.9)	53 (48.6)	18 611 (56.6)	1892 (69.8)	161 (60.8)
GINA Step, n (%)							
None	636 (0.4)	NA	NA	NA	199 (0.6)	NA	NA
1	68 268 (45.0)	NA	NA	NA	8256 (25.1)	NA	NA
2	34 404 (22.7)	NA	NA	NA	6966 (21.2)	NA	NA
3	30 155 (19.9)	NA	NA	NA	14 761 (44.9)	NA	NA
4	17 905 (11.8)	17 905 (99.0)	2067 (98.5)	106 (97.2)	2704 (8.2)	2704 (99.7)	262 (98.9)
5	181 (0.1)	181 (1.0)	32 (1.5)	3 (2.8)	7 (<0.1)	7 (0.3)	3 (1.1)
Severe asthma exacerbations per patient during the baseline period, by type,^b^ median (range)							
All types	0 (0, 31)	0 (0, 21)	2 (2, 21)	2 (2, 7)	0 (0, 15)	0 (0, 15)	2 (2, 15)
OCS‐defined exacerbations	0 (0, 7)	0 (0, 7)	2 (0, 7)	2 (0, 6)	0 (0, 13)	0 (0, 13)	2 (0, 13)
Exacerbations resulting in ED visit	0 (0, 31)	0 (0, 21)	0 (0, 21)	0 (0, 5)	0 (0, 12)	0 (0, 12)	0 (0, 12)
Exacerbations resulting in hospitalization	0 (0, 4)	0 (0, 4)	0 (0, 4)	0 (0, 4)	0 (0, 17)	0 (0, 17)	0 (0, 17)
Number of severe asthma exacerbations experienced during the baseline period, all types, n (%)							
0	114 477 (75.5)	11 580 (64.0)	NA	NA	28 810 (87.6)	1941 (71.8)	NA
1	29 565 (19.5)	4407 (24.4)	NA	NA	3263 (9.9)	499 (18.4)	NA
2	5660 (3.7)	1415 (7.8)	1415 (67.4)	71 (65.1)	583 (1.8)	148 (5.5)	148 (55.8)
3	1357 (0.9)	461 (2.5)	461 (22.0)	20 (18.3)	153 (0.5)	66 (2.4)	66 (24.9)
≥4	490 (0.3)	223 (1.2)	223 (10.6)	18 (16.5)	84 (0.3)	51 (1.9)	51 (19.2)
Number of severe OCS‐defined exacerbations^c^ experienced during the baseline period, n (%)							
0	117 673 (77.6)	12 045 (66.6)	67 (3.2)	2 (1.8)	29 529 (89.8)	2072 (76.4)	26 (9.8)
1	27 577 (18.2)	4227 (23.4)	218 (10.4)	12 (11.0)	2698 (8.2)	428 (15.8)	56 (21.1)
≥2	6299 (4.2)	1814 (10.0)	1814 (86.4)	95 (87.2)	666 (2.0)	211 (7.8)	183 (69.1)
Number of severe exacerbations resulting in ED visit experienced during the baseline period, n (%)							
0	144 940 (95.6)	16 897 (93.4)	1474 (70.2)	74 (67.9)	32 261 (98.1)	2563 (94.5)	184 (69.4)
1	5809 (3.8)	971 (5.4)	407 (19.4)	21 (19.3)	529 (1.6)	109 (4.0)	42 (15.8)
≥2	800 (0.5)	218 (1.2)	218 (10.4)	14 (12.8)	103 (0.3)	39 (1.4)	39 (14.7)
Number of severe exacerbations resulting in hospitalization experienced during the baseline period, n (%)							
0	149 749 (98.8)	17 594 (97.3)	1813 (86.4)	95 (87.2)	32 204 (97.9)	2523 (93.1)	147 (55.5)
1	1657 (1.1)	438 (2.4)	232 (11.1)	7 (6.4)	552 (1.7)	126 (4.6)	60 (22.6)
≥2	143 (0.1)	54 (0.3)	54 (2.6)	7 (6.4)	137 (0.4)	62 (2.3)	58 (21.9)
Patients with blood eosinophil measurements, n (%)	10 176 (6.7)	1403 (7.8)	177 (8.4)	109 (100)	289 (0.9)	28 (1.0)	8 (3.0)
Eosinophil counts, cells/µL, median (IQR)^d^	200 (100‐400)	200 (100‐394)	200 (100‐447)	400 (203‐600)	338 (163‐560)	380 (170‐790)	…
Eosinophil category, n (%)^d^							
<150 cells/µL	3885 (2.6)	573 (3.2)	68 (3.2)	NA	70 (0.2)	6 (0.2)	…
≥150 to <300 cells/µL	2346 (1.5)	311 (1.7)	32 (1.5)	32 (29.4)	59 (0.2)	5 (0.2)	…
≥300 cells/µL	3945 (2.6)	519 (2.9)	77 (3.7)	77 (70.6)	160 (0.5)	17 (0.6)	…

*Note*: US‐treated asthma group: n = 151 549, US severe asthma group: n = 18 086, US severe refractory asthma group: n = 2099, US severe refractory eosinophilic asthma group: n = 109, England‐treated asthma group: n = 32 893, England severe asthma group: n = 2711, England severe refractory asthma group: n = 265. In the England population data for the severe refractory eosinophilic asthma group are not reported owing to the small sample size.

Abbreviations: ED, emergency department; GINA, Global Initiative for Asthma; IQR, interquartile range; NA, not applicable; OCS, oral corticosteroid.

^a^ Data for the severe refractory eosinophilic asthma group (n = 8) are not reported owing to the small sample size.

^b^ The maximum number of exacerbations within the “all‐type” category may be lower than the maximum estimated when considering each type of exacerbation separately. This is due to the requirement for a 7 d gap between each unique exacerbation.

^c^ Defined as OCS prescription (England population) or any OCS prescription equivalent to prednisone 20 mg/d for 3 to 28 d (US population) recorded within 1 wk before or 1 wk following an asthma diagnosis code.

^d^ Analyses of eosinophil counts were not performed for the severe refractory asthma group in England owing to the small sample size (n = 8).

### 3.2 Exacerbations during follow‐up

**Figure 2:**
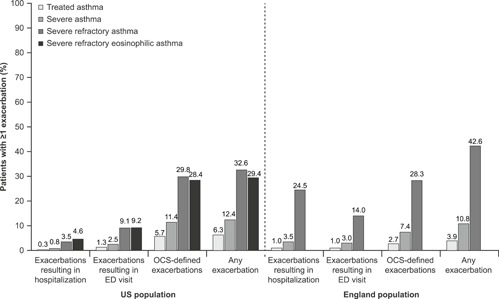
Exacerbations during the follow‐up period. US‐treated asthma group: n = 151 549, US severe asthma group: n = 18 086, US severe refractory asthma group: n = 2099, US severe refractory eosinophilic asthma group: n = 109, England‐treated asthma group: n = 32 893, England severe asthma group: n = 2711, England severe refractory asthma group: n = 265. Asthma exacerbations were defined as any exacerbation with asthma in the primary position for exacerbations that resulted in hospitalization or an emergency department (ED) visit. Oral corticosteroid (OCS)‐defined exacerbations were defined as OCS prescription (England population) or any OCS prescription equivalent to prednisone 20 mg/d for 3 to 28 days (US population) recorded within 1 week before or 1 week following an asthma diagnosis code. In the England population, data for the severe refractory eosinophilic asthma group are not reported owing to the small sample size

In both the US and England, the proportion of patients experiencing ≥1 exacerbation during the follow‐up period was lowest in the treated asthma group for any exacerbation type and increased with asthma severity (Figure 2). For example, in the treated asthma group, 6.3% of the US population and 3.9% of the England population experienced ≥1 exacerbation during follow‐up; in the severe refractory asthma group, this increased to 32.6% and 42.6%, respectively (Figure 2). In the treated asthma group, the proportion of patients experiencing ≥1 exacerbation resulting in hospitalization was 0.3% in the US population and 1.0% in the England population, increasing to 3.5% and 24.5%, respectively, in the severe refractory asthma group (Figure 2). In both populations, OCS‐defined exacerbations were the most common exacerbation type. Furthermore, the number of exacerbations experienced during follow‐up increased with increasing GINA step (Table S2) and with increasing exacerbation frequency in the baseline period (Table S3).

### 3.3 HCRU in the 30 days following the first severe asthma exacerbation

**Figure 3:**
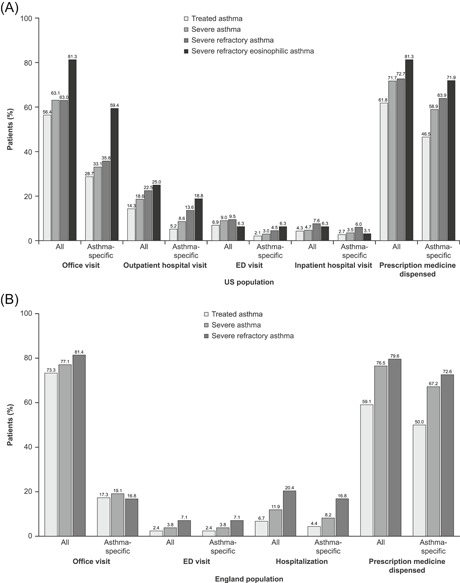
Health care resource utilization (HCRU) in the 30 days following the first severe asthma exacerbation in (A) the US and (B) England. US‐treated asthma group: n = 151 549, US severe asthma group: n = 18 086, US severe refractory asthma group: n = 2099, US severe refractory eosinophilic asthma group: n = 109, England‐treated asthma group: n = 32 893, England severe asthma group: n = 2711, England severe refractory asthma group: n = 265. In the England population, data for the severe refractory eosinophilic asthma group are not reported owing to the small sample size. ED, emergency department

In the US, all‐cause and asthma‐related HCRU were lower in the treated asthma group and increased with disease severity (Figure 3A). For example, 2.7% and 5.2% of patients with treated asthma, 3.5% and 8.6% with severe asthma, and 6.0% and 13.6% with severe refractory asthma had ≥1 asthma‐specific inpatient or outpatient hospitalization visit, respectively. Similarly, the proportion of patients with ≥1 asthma‐specific ED visit ranged from 2.1% among patients with treated asthma to 4.5% in patients with severe refractory asthma (Figure 3A).

Similar trends were seen in the population from England (Figure 3B). For example, 4.4% of patients with treated asthma, 8.2% with severe asthma, and 16.8% with severe refractory asthma had ≥1 asthma‐specific hospitalization visit. In addition, the proportion of patients with ≥1 asthma‐specific ED visit ranged from 2.4% in the treated asthma group to 7.1% in the severe refractory asthma group (Figure 3B). There was also a trend toward higher all‐cause and asthma‐related HCRU with an increasing number of exacerbations in the baseline period (Table S4).

### 3.4 Hospital readmissions within 30 or 90 days following the first severe asthma exacerbation

**Figure 4:**
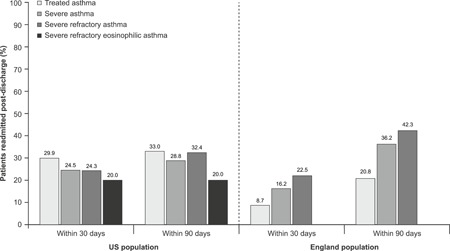
Readmissions following the first‐hospital discharge. US‐treated asthma group: n = 151 549, US severe asthma group: n = 18 086, US severe refractory asthma group: n = 2099, US severe refractory eosinophilic asthma group: n = 109, England‐treated asthma group: n = 32 893, England severe asthma group: n = 2711, England severe refractory asthma group: n = 265. In the England population, data for the severe refractory eosinophilic asthma group are not reported owing to the small sample size

In England, hospital readmissions within 30 or 90 days following the first severe asthma exacerbation discharge were lowest in the treated asthma group (8.7% and 20.8%, respectively) and increased with disease severity to 22.5% and 42.3% in the severe refractory asthma group (Figure 4). In the US, no such pattern was observed, and the proportion of patients with readmissions ranged from 24.3% to 29.9% for readmissions within 30 days and 28.8% to 33.0% for those within 90 days (Figure 4).

### 3.5 Asthma treatments within 30 days following the first hospital discharge after an asthma exacerbation

Overall, in both the US and England, short‐acting beta‐agonists (SABAs) were the most commonly prescribed asthma medication within 30 days following the first hospital discharge after an asthma exacerbation in all groups (Figure S1). The proportion of patients receiving SABA prescriptions was lower in the US (50.8%‐61.1% across severity groups) than in England (84.2%‐89.1%). In the US, leukotriene receptor antagonists (LTRAs) and OCS were prescribed in 40.7% to 60.0% and 41.7% to 60.0% of patients, respectively, within 30 days of first hospital discharge, followed by ICS (0.0%‐33.3%) and ICS‐long‐acting beta‐agonist (LABA) (20.0%‐35.2%) (Figure S1A). In England, ICS‐LABA was prescribed in 39.1% to 61.8% of patients within 30 days of first‐hospital discharge, followed by OCS (23.3%‐32.7%), ICS (9.1%‐29.8%), and LABA (3.3%‐6.2%) (Figure S1B).

## 4 DISCUSSION

In this retrospective, real‐world study of data from pediatric patients from two large databases from England and the US, exacerbation frequency and HCRU tended to increase with asthma severity, indicating that disease burden increases with disease severity. The overall trend for a higher burden of exacerbations and HCRU in pediatric patients with more severe asthma is consistent with trends reported in adults.^7^, ^9^, ^10^, ^11^, ^12^ Moreover, our findings are consistent with those from another study conducted in adolescents (aged ≥ 12 years) and adult patients with asthma,^8^ thus highlighting the need for improved disease management in both pediatric and adult patients with severe disease. As expected, across all groups, asthma exacerbations during the follow‐up period were more frequent if patients had more exacerbations in the baseline period. Previous studies have also reported an association between asthma exacerbation history and future exacerbation risk.^8^, ^21^, ^22^, ^23^

While our results highlight the proportions of patients in the different asthma groups that have unmet treatment needs, our findings also indicate some positive observations with regard to current asthma management practices. For example, of the patients with severe refractory asthma who were required by definition to have ≥2 baseline exacerbations, only around 32% of patients in the US and 42% of patients in England had ≥1 exacerbation (any type) during the follow‐up period. These results support the current asthma management strategies, demonstrating that they are effective in preventing the worsening of symptoms in more than half of these patients. However, future research should focus on the unmet needs of the remaining patients who do not benefit from their current asthma management.

Our study demonstrated that there were some differences in HCRU between patients in the US and England, despite the similar methodology used for analyzing both datasets (including similar definitions to define disease severity groups). This is to be expected as the two populations were from different datasets coming from different geographic settings and were exposed to different health care systems, which could lead to different clinical practices and different asthma management regimens. First, the proportion of patients experiencing exacerbations resulting in hospitalization or an ED visit during the follow‐up period was generally higher in England than in the US. Second, readmission within 90 days of a severe exacerbation was also higher in England than in the US, as was the proportion of patients with ≥1 office visit, and this may be attributed to differences in access and affordability of health care between these two countries. Third, asthma‐specific office visits were higher in the US than in England, and it is possible that general practitioner visits in England may have covered issues in addition to asthma, leading to an underestimation in the number of asthma‐specific visits. Fourth, all‐cause and asthma‐specific hospitalizations were higher in England than in the US. Finally, there were also differences in the proportions of patients on LABA monotherapy (almost none in the US), and higher LTRA and OCS use were seen in the US compared with England possibly due to different concerns about safety, leading to different country practices.

In this study, few patients had full blood eosinophil count data available, making it difficult to clearly identify those with eosinophilic asthma. It is possible that pediatricians may consider it unhelpful to measure blood eosinophil counts in pediatric patients, unlike in adults in whom blood testing is routinely performed. This is likely to explain the low patient numbers in the severe refractory eosinophilic asthma subgroup in our study. The number of patients with blood eosinophil tests recorded was higher in the US (6.7%) than in England (0.9%), perhaps owing to differences in data sources or physician practices between the two countries. Indeed, if we are to better understand severe refractory asthma and severe refractory eosinophilic asthma in the future, we need to identify those with elevated eosinophils across countries.^24^, ^25^

There are several limitations that should be taken into account when considering the results from this analysis. First, medication usage to assess GINA severity was determined from the prescription information in the databases rather than clinical assessment. Second, the frequency of OCS‐defined exacerbations may have been underestimated, as some patients had a supply of OCS at home (particularly those who experienced frequent exacerbations) and because the 20 mg/d (prednisone equivalent) cut‐off used in the US study to categorize OCS‐defined exacerbations may have excluded younger pediatric patients prescribed OCS at a lower dose. Third, the lack of available patient‐reported outcome data or lung function measurements was also an issue. Finally, findings from this study may not be generalizable to other countries where practices differ and patients with asthma may have different disease causations, history, and progression that will impact their treatment regimen.

In conclusion, our results indicate that in pediatric patients with asthma studied, the burden of asthma tended to be higher as disease severity increased. In addition, our data provide an insight into the effectiveness of current asthma management practices in the US and England and point to subsets of patients with severe disease who may have an unmet need for effective treatment to minimize the risk of exacerbations and optimize clinical outcomes.

## CONFLICT OF INTERESTS

MG, WL, and MKVD are employees of GlaxoSmithKline (GSK) and hold stocks/shares. BN, JC, and AS are employees of Evidera, a consulting company that received funding from GSK for the research presented in this manuscript. TH was an employee of GSK during the study and is now an employee of AstraZeneca. LE was an employee of GSK during the study and is now an employee of ViiV Healthcare Limited under a GSK contract and holds stocks/shares in GSK.

## AUTHOR CONTRIBUTIONS

MG, TH, LE, and MKVD were involved in the conception or design of the study, acquisition of data, and data analysis or interpretation. WL contributed to data analysis and interpretation. BN, JC, and AS were involved in the conception or design of the study and data analysis and interpretation. All authors critically revised the manuscript for intellectual content, gave final approval of the version to be published, and agreed to be accountable for all aspects of the work.

## Supporting information

Supporting information

## Data Availability

GlaxoSmithKline (GSK) makes available anonymized individual participant data and associated documents from interventional clinical studies that evaluate medicines, upon approval of proposals submitted to http://www.clinicalstudydatarequest.com. To access data for other types of GSK sponsored research, for study documents without patient‐level data and for clinical studies not listed, please submit an inquiry via the website. The data that support the findings of this study are available from UK CPRD. Researchers can apply to access CPRD data; access is conditional on approval by the independent scientific advisory committee.
