# Supplementary material for: Burden of asthma exacerbations and health care utilization in pediatric patients with asthma in the US and England
Source: Immun Inflamm Dis. 2020 Mar 28;8(2):236–45. doi: 10.1002/iid3.299 (PMC7212194; doi:10.1002/iid3.299)
Supplement: Supplementary file 1 — Supporting information [file IID3-8-236-s001.docx]

**Supplementary Materials**

**Burden of asthma exacerbations and healthcare utilization in pediatric patients with asthma in the US and England**

**Gokhale *et al*.**

**Corresponding author: Melissa K. Van Dyke, Epidemiology, GSK, 1250 South Collegeville Road, Upper Providence, PA 19426, USA. Tel: +1 215-565-6802. Email:** [**melissa.k.van-dyke@gsk.com**](mailto:melissa.k.van-dyke@gsk.com)

**Contents**

**Table S1.** Asthma diagnosis codes

**Table S2.** Exacerbations during the follow-up period by GINA step

**Table S3.** Exacerbations during the follow-up period by number of exacerbations during the baseline period

**Table S4.** HCRU, by exacerbation history, in the 30 days following the first severe asthma exacerbation

**Figure S1.** Treatments prescribed within 30 days following the first hospital discharge after an asthma exacerbation in A) the US and b) England

**Table S1.** Asthma diagnosis codes

| **ICD-9-CM code** | **ICD-10 code** | **Read code** | **Classification** |
| --- | --- | --- | --- |
| **US** | | | |
| 493 | J45 | **-** | Asthma |
| 493.00 | J45.20 | **-** | Mild intermittent asthma, uncomplicated |
| 493.02 | J45.21 | **-** | Extrinsic asthma with (acute) exacerbation |
| 493.12 | J45.21 | **-** | Mild intermittent asthma with (acute) exacerbation |
| 493.00 | J45.30 | **-** | Mild persistent asthma, uncomplicated |
| 493.00 | J45.40 | **-** | Moderate persistent asthma, uncomplicated |
| 493.00 | J45.50 | **-** | Severe persistent asthma, uncomplicated |
| 493.92 | J45.901 | **-** | Asthma, unspecified type, with (acute) exacerbation |
| 493.90 | J45.909 | **-** | Unspecified asthma, uncomplicated |
| 493.90 | J45.998 | **-** | Other asthma |
| **England** | | | |
| **-** | J45 | **-** | Asthma |
| **-** | J46 | **-** | Status asthmaticus |
| **-** | **-** | 102.00 | Asthma confirmed |
| **-** | **-** | 2126200 | Asthma resolved |
| **-** | **-** | 212G.00 | Asthma resolved |
| **-** | **-** | 663V100 | Mild asthma |
| **-** | **-** | 663V200 | Moderate asthma |
| **-** | **-** | 663V300 | Severe asthma |
| **-** | **-** | 8H2P.00 | Emergency admission, asthma |
| **-** | **-** | H33.00 | Asthma |
| **-** | **-** | H333.00 | Acute exacerbation of asthma |
| **-** | **-** | H33z.00 | Asthma unspecified |
| **-** | **-** | H33z011 | Severe asthma attack |
| **-** | - | H33z100 | Asthma attack |

CM, clinical modification; ICD, International Classification of Diseases.

**Table S2.** Exacerbations during the follow-up period by GINA step

|  | **Treated asthma** | | | | | | **Severe asthma** | | **Severe refractory asthma** | | **Severe refractory eosinophilic asthma** | |
| --- | --- | --- | --- | --- | --- | --- | --- | --- | --- | --- | --- | --- |
|  | **GINA Step** | | | | | | | | | | | |
|  | **None** | **1** | **2** | **3** | **4** | **5** | **4** | **5** | **4** | **5** | **4** | **5** |
| **US population** | | | | | | | | | | | | |
| **All exacerbations, n (%)** | | | | | | | | | | | | |
| **0** | 562 (88.4) | 65,268 (95.6) | 32,391 (94.1) | 27,874 (92.4) | 15,702 (87.7) | 148 (81.8) | 15,702 (87.7) | 148 (81.8) | 1,394 (67.4) | 21 (65.6) | 75 (70.8) | 2 (66.7) |
| **1** | 58 (9.1) | 2,545 (3.7) | 1,697 (4.9) | 1,890 (6.3) | 1,682 (9.4) | 22 (12.2) | 1,682 (9.4) | 22 (12.2) | 412 (19.9) | 7 (21.9) | 20 (18.9) | 1 (33.3) |
| **2** | 14 (2.2) | 358 (0.5) | 242 (0.7) | 312 (1.0) | 367 (2.0) | 7 (3.9) | 367 (2.0) | 7 (3.9) | 169 (8.2) | 2 (6.3) | 6 (5.7) | 0 (0.0) |
| **3** | 2 (0.3) | 74 (0.1) | 45 (0.1) | 58 (0.2) | 100 (0.6) | 1 (0.6) | 100 (0.6) | 1 (0.6) | 49 (2.4) | 0 (0.0) | 2 (1.9) | 0 (0.0) |
| **≥4** | 0 (0.0) | 23 (0.0) | 29 (0.1) | 21 (0.1) | 54 (0.3) | 3 (1.7) | 54 (0.3) | 3 (1.7) | 43 (2.1) | 2 (6.3) | 3 (2.8) | 0 (0.0) |
| **Exacerbations resulting in hospitalization, n (%)** | | | | | | | | | | | | |
| **0** | 631 (99.2) | 68,122 (99.8) | 34,313 (99.7) | 30,051 (99.7) | 17,770 (99.2) | 177 (97.8) | 17,770 (99.2) | 177 (97.8) | 1,996 (96.6) | 29 (90.6) | 101 (95.3) | 3 (100.0) |
| **1** | 4 (0.6) | 135 (0.2) | 87 (0.3) | 94 (0.3) | 121 (0.7) | 2 (1.1) | 121 (0.7) | 2 (1.1) | 59 (2.9) | 1 (3.1) | 5 (4.7) | 0 (0.0) |
| **≥2** | 1 (0.2) | 11 (0.0) | 4 (0.0) | 10 (0.0) | 14 (0.1) | 2 (1.1) | 14 (0.1) | 2 (1.1) | 12 (0.6) | 2 (6.3) | 0 (0.0) | 0 (0.0) |
| **Exacerbations resulting in ED visit, n (%)** | | | | | | | | | | | | |
| **0** | 621 (97.6) | 67,569 (99.0) | 34,060 (99.0) | 29,723 (98.6) | 17,466 (97.5) | 172 (95.0) | 17,466 (97.5) | 172 (95.0) | 1,879 (90.9) | 28 (87.5) | 96 (90.6) | 3 (100.0) |
| **1** | 11 (1.7) | 631 (0.9) | 308 (0.9) | 399 (1.3) | 358 (2.0) | 6 (3.3) | 358 (2.0) | 6 (3.3) | 135 (6.5) | 2 (6.3) | 6 (5.7) | 0 (0.0) |
| **≥2** | 4 (0.6) | 68 (0.1) | 36 (0.1) | 33 (0.1) | 81 (0.5) | 3 (1.7) | 81 (0.5) | 3 (1.7) | 53 (2.6) | 2 (6.3) | 4 (3.8) | 0 (0.0) |
| **OCS-defined exacerbations, n (%)** | | | | | | | | | | | | |
| **0** | 572 (89.9) | 65,658 (96.2) | 32,577 (94.7) | 28,082 (93.1) | 15,880 (88.7) | 153 (84.5) | 15,880 (88.7) | 153 (84.5) | 1,451 (70.2) | 23 (71.9) | 76 (71.7) | 2 (66.7) |
| **1** | 53 (8.3) | 2,259 (3.3) | 1,556 (4.5) | 1,752 (5.8) | 1,602 (8.9) | 20 (11.0) | 1,602 (8.9) | 20 (11.0) | 411 (19.9) | 6 (18.8) | 22 (20.8) | 1 (33.3) |
| **≥2** | 11 (1.7) | 351 (0.5) | 271 (0.8) | 321 (1.1) | 423 (2.4) | 8 (4.4) | 423 (2.4) | 8 (4.4) | 205 (9.9) | 3 (9.4) | 8 (7.5) | 0 (0.0) |
| **England population** | | | | | | | | | | | | |
| **All exacerbations, n (%)** | | | | | | | | | | | | |
| **0** | 193 (97.0) | 8,083 (97.9) | 6,770 (97.2) | 14,143 (95.8) | 2,413 (89.2) | 5 (71.4) | 2,413 (89.2) | 5 (71.4) | 151 (57.6) | 1 (33.3) | NA | NA |
| **1** | 5 (2.5) | 148 (1.8) | 168 (2.4) | 515 (3.5) | 205 (7.6) | 1 (14.3) | 205 (7.6) | 1 (14.3) | 50 (19.1) | 1 (33.3) | NA | NA |
| **2** | 1 (0.5) | 17 (0.2) | 25 (0.4) | 76 (0.5) | 44 (1.6) | 1 (14.3) | 44 (1.6) | 1 (14.3) | 23 (8.8) | 1 (33.3) | NA | NA |
| **3** | 0 (0.0) | 5 (0.1) | 3 (0.0) | 20 (0.1) | 15 (0.6) | 0 (0.0) | 15 (0.6) | 0 (0.0) | 12 (4.6) | 0 (0.0) | NA | NA |
| **≥4** | 0 (0.0) | 3 (0.0) | 0 (0.0) | 7 (0.0) | 27 (1.0) | 0 (0.0) | 27 (1.0) | 0 (0.0) | 26 (9.9) | 0 (0.0) | NA | NA |
| **Exacerbations resulting in hospitalization, n (%)** | | | | | | | | | | | | |
| **0** | 195 (98.0) | 8,215 (99.5) | 6,929 (99.5) | 14,617 (99.0) | 2,610 (96.5) | 5 (71.4) | 2,610 (96.5) | 5 (71.4) | 199 (76.0) | 1 (33.3) | NA | NA |
| **1** | 4 (2.0) | 30 (0.4) | 34 (0.5) | 116 (0.8) | 54 (2.0) | 2 (28.6) | 54 (2.0) | 2 (28.6) | 30 (11.5) | 2 (66.7) | NA | NA |
| **≥2** | 0 (0.0) | 11 (0.1) | 3 (0.0) | 28 (0.2) | 40 (1.5) | 0 (0.0) | 40 (1.5) | 0 (0.0) | 33 (12.6) | 0 (0.0) | NA | NA |
| **Exacerbations resulting in ED visit, n (%)** | | | | | | | | | | | | |
| **0** | 198 (99.5) | 8,218 (99.5) | 6,906 (99.1) | 14,602 (98.9) | 2,623 (97.0) | 6 (85.7) | 2,623 (97.0) | 6 (85.7) | 226 (86.3) | 2 (66.7) | NA | NA |
| **1** | 1 (0.5) | 35 (0.4) | 55 (0.8) | 136 (0.9) | 63 (2.3) | 1 (14.3) | 63 (2.3) | 1 (14.3) | 20 (7.6) | 1 (33.3) | NA | NA |
| **≥2** | 0 (0.0) | 3 (0.0) | 5 (0.1) | 23 (0.2) | 18 (0.7) | 0 (0.0) | 18 (0.7) | 0 (0.0) | 16 (6.1) | 0 (0.0) | NA | NA |
| **OCS-defined exacerbations, n (%)** | | | | | | | | | | | | |
| **0** | 197 (99.0) | 8,128 (98.4) | 6,838 (98.2) | 14,330 (97.1) | 2,504 (92.6) | 6 (85.7) | 2,504 (92.6) | 6 (85.7) | 188 (71.8) | 2 (66.7) | NA | NA |
| **1** | 1 (0.5) | 113 (1.4) | 108 (1.6) | 363 (2.5) | 143 (5.3) | 1 (14.3) | 143 (5.3) | 1 (14.3) | 39 (14.9) | 1 (33.3) | NA | NA |
| **≥2** | 1 (0.5) | 15 (0.2) | 20 (0.3) | 68 (0.5) | 57 (2.1) | 0 (0.0) | 57 (2.1) | 0 (0.0) | 35 (13.4) | 0 (0.0) | NA | NA |

ED, emergency department; GINA, Global Initiative for Asthma; NA, not applicable; OCS, oral corticosteroid.

Asthma exacerbations were defined as any exacerbation with asthma in the primary position for exacerbations that resulted in hospitalization or an ED visit. OCS-defined exacerbations were defined as OCS prescription (England population) or any OCS prescription equivalent to prednisone 20 mg/day for 3–28 days (US population) recorded within 1 week prior to or 1 week following an asthma diagnosis code. In the England population, data for the severe refractory eosinophilic asthma group are not reported owing to the small sample size.

**Table S3.** Exacerbations during the follow-up period by number of exacerbations during the baseline period

|  | **Treated asthma** | | | | | **Severe asthma** | | | | | **Severe refractory asthma cohort** | | | **Severe refractory eosinophilic asthma** | | |
| --- | --- | --- | --- | --- | --- | --- | --- | --- | --- | --- | --- | --- | --- | --- | --- | --- |
|  | **Number of exacerbations during baseline period** | | | | | | | | | | | | | | | |
|  | **None** | **1** | **2** | **3** | **≥4** | **None** | **1** | **2** | **3** | **≥4** | **2** | **3** | **≥4** | **2** | **3** | **≥4** |
| **US population** | | | | | |  | | | | |  | | |  | | |
| **All exacerbations, n (%)** | | | | | | | | | | | | | | | | |
| **0** | 109,922 (96.0) | 26,467 (89.5) | 4,448 (78.6) | 879 (64.8) | 229 (46.7) | 10,749 (92.8) | 3,686 (83.6) | 1,045 (73.9) | 276 (59.9) | 94 (42.2) | 1,045 (73.9) | 276 (59.9) | 94 (42.2) | 55 (77.5) | 15 (75.0) | 7 (38.9) |
| **1** | 4,010 (3.5) | 2,569 (8.7) | 902 (15.9) | 303 (22.3) | 110 (22.4) | 711 (6.1) | 574 (13.0) | 257 (18.2) | 114 (24.7) | 48 (21.5) | 257 (18.2) | 114 (24.7) | 48 (21.5) | 12 (16.9) | 4 (20.0) | 5 (27.8) |
| **2** | 472 (0.4) | 409 (1.4) | 221 (3.9) | 124 (9.1) | 74 (15.1) | 98 (0.8) | 105 (2.4) | 80 (5.7) | 50 (10.8) | 41 (18.4) | 80 (5.7) | 50 (10.8) | 41 (18.4) | 3 (4.2) | 0 (0.0) | 3 (16.7) |
| **3** | 63 (0.1) | 94 (0.3) | 62 (1.1) | 31 (2.3) | 30 (6.1) | 18 (0.2) | 34 (0.8) | 23 (1.6) | 12 (2.6) | 14 (6.3) | 23 (1.6) | 12 (2.6) | 14 (6.3) | 1 (1.4) | 0 (0.0) | 1 (5.6) |
| **≥4** | 10 (0.0) | 26 (0.1) | 27 (0.5) | 20 (1.5) | 47 (9.6) | 4 (0.0) | 8 (0.2) | 10 (0.7) | 9 (2.0) | 26 (11.7) | 10 (0.7) | 9 (2.0) | 26 (11.7) | 0 (0.0) | 1 (5.0) | 2 (11.1) |
| **Exacerbations resulting in hospitalization, n (%)** | | | | | | | | | | | | | | | | |
| **0** | 114,277 (99.8) | 29,442 (99.6) | 5,580 (98.6) | 1,316 (97.0) | 449 (91.6) | 11,544 (99.7) | 4,378 (99.3) | 1,385 (97.9) | 442 (95.9) | 198 (88.8) | 1,385 (97.9) | 442 (95.9) | 198 (88.8) | 69 (97.2) | 20 (100.0) | 15 (83.3) |
| **1** | 187 (0.2) | 118 (0.4) | 71 (1.3) | 37 (2.7) | 30 (6.1) | 34 (0.3) | 29 (0.7) | 24 (1.7) | 18 (3.9) | 18 (8.1) | 24 (1.7) | 18 (3.9) | 18 (8.1) | 2 (2.8) | 0 (0.0) | 3 (16.7) |
| **≥2** | 13 (0.0) | 5 (0.0) | 9 (0.2) | 4 (0.3) | 11 (2.2) | 2 (0.0) | 0 (0.0) | 6 (0.4) | 1 (0.2) | 7 (3.1) | 6 (0.4) | 1 (0.2) | 7 (3.1) | 0 (0.0) | 0 (0.0) | 0 (0.0) |
| **Exacerbations resulting in ED visit, n (%)** | | | | | | | | | | | | | | | | |
| **0** | 113,682 (99.3) | 28,934 (97.9) | 5,392 (95.3) | 1,224 (90.2) | 379 (77.3) | 11,450 (98.9) | 4,281 (97.1) | 1,331 (94.1) | 410 (88.9) | 166 (74.4) | 1,331 (94.1) | 410 (88.9) | 166 (74.4) | 67 (94.4) | 18 (90.0) | 14 (77.8) |
| **1** | 744 (0.6) | 581 (2.0) | 219 (3.9) | 107 (7.9) | 62 (12.7) | 119 (1.0) | 108 (2.5) | 63 (4.5) | 40 (8.7) | 34 (15.2) | 63 (4.5) | 40 (8.7) | 34 (15.2) | 2 (2.8) | 2 (10.0) | 2 (11.1) |
| **≥2** | 51 (0.0) | 50 (0.2) | 49 (0.9) | 26 (1.9) | 49 (10.0) | 11 (0.1) | 18 (0.4) | 21 (1.5) | 11 (2.4) | 23 (10.3) | 21 (1.5) | 11 (2.4) | 23 (10.3) | 2 (2.8) | 0 (0.0) | 2 (11.1) |
| **OCS-defined exacerbations, n (%)** | | | | | | | | | | | | | | | | |
| **0** | 110,376 (96.4) | 26,785 (90.6) | 4,560 (80.6) | 936 (69.0) | 265 (54.1) | 10,818 (93.4) | 3,741 (84.9) | 1,070 (75.6) | 298 (64.6) | 106 (47.5) | 1,070 (75.6) | 298 (64.6) | 106 (47.5) | 55 (77.5) | 16 (80.0) | 7 (38.9) |
| **1** | 3,653 (3.2) | 2,344 (7.9) | 855 (15.1) | 279 (20.6) | 111 (22.7) | 663 (5.7) | 542 (12.3) | 260 (18.4) | 108 (23.4) | 49 (22.0) | 260 (18.4) | 108 (23.4) | 49 (22.0) | 14 (19.7) | 3 (15.0) | 6 (33.3) |
| **≥2** | 448 (0.4) | 436 (1.5) | 245 (4.3) | 142 (10.5) | 114 (23.3) | 99 (0.9) | 124 (2.8) | 85 (6.0) | 55 (11.9) | 68 (30.5) | 85 (6.0) | 55 (11.9) | 68 (30.5) | 2 (2.8) | 1 (5.0) | 5 (27.8) |
| **England population** | | | | | |  | | | | |  | | |  | | |
| **All exacerbations, n (%)** | | | | | | | | | | | | | | | | |
| **0** | 28,146 (97.7) | 2,903 (89.0) | 445 (76.3) | 84 (54.9) | 29 (34.5) | 1,852 (95.1) | 414 (83.0) | 104 (70.3) | 31 (47.0) | 17 (33.3) | 104 (70.3) | 31 (47.0) | 17 (33.3) | NA | NA | NA |
| **1** | 606 (2.1) | 284 (8.7) | 99 (17.0) | 37 (24.2) | 16 (19.0) | 86 (4.4) | 69 (13.8) | 28 (18.9) | 17 (25.8) | 6 (11.8) | 28 (18.9) | 17 (25.8) | 6 (11.8) | NA | NA | NA |
| **2** | 48 (0.2) | 65 (2.0) | 25 (4.3) | 15 (9.8) | 11 (13.1) | 8 (0.4) | 13 (2.6) | 9 (6.1) | 7 (10.6) | 8 (15.7) | 9 (6.1) | 7 (10.6) | 8 (15.7) | NA | NA | NA |
| **3** | 9 (0.0) | 7 (0.2) | 6 (1.0) | 10 (6.5) | 11 (13.1) | 1 (0.1) | 2 (0.4) | 1 (0.7) | 5 (7.6) | 6 (11.8) | 1 (0.7) | 5 (7.6) | 6 (11.8) | NA | NA | NA |
| **≥4** | 1 (0.0) | 4 (0.1) | 8 (1.4) | 7 (4.6) | 17 (20.2) | 0 (0.0) | 1 (0.2) | 6 (4.1) | 6 (9.1) | 14 (27.5) | 6 (4.1) | 6 (9.1) | 14 (27.5) | NA | NA | NA |
| **Exacerbations resulting in hospitalization, n (%)** | | | | | | | | | | | | | | | | |
| **0** | 28,701 (99.6) | 3,172 (97.2) | 534 (91.6) | 119 (77.8) | 45 (53.6) | 1,930 (99.1) | 485 (97.2) | 128 (86.5) | 48 (72.7) | 24 (47.1) | 128 (86.5) | 48 (72.7) | 24 (47.1) | NA | NA | NA |
| **1** | 99 (0.3) | 70 (2.1) | 35 (6.0) | 22 (14.4) | 14 (16.7) | 14 (0.7) | 10 (2.0) | 13 (8.8) | 9 (13.6) | 10 (19.6) | 13 (8.8) | 9 (13.6) | 10 (19.6) | NA | NA | NA |
| **≥2** | 10 (0.0) | 21 (0.6) | 14 (2.4) | 12 (7.8) | 25 (29.8) | 3 (0.2) | 4 (0.8) | 7 (4.7) | 9 (13.6) | 17 (33.3) | 7 (4.7) | 9 (13.6) | 17 (33.3) | NA | NA | NA |
| **Exacerbations resulting in ED visit, n (%)** | | | | | | | | | | | | | | | | |
| **0** | 28,651 (99.4) | 3,169 (97.1) | 545 (93.5) | 124 (81.0) | 64 (76.2) | 1,927 (99.0) | 474 (95.0) | 136 (91.9) | 52 (78.8) | 40 (78.4) | 136 (91.9) | 52 (78.8) | 40 (78.4) | NA | NA | NA |
| **1** | 148 (0.5) | 79 (2.4) | 32 (5.5) | 22 (14.4) | 10 (11.9) | 20 (1.0) | 23 (4.6) | 9 (6.1) | 8 (12.1) | 4 (7.8) | 9 (6.1) | 8 (12.1) | 4 (7.8) | NA | NA | NA |
| **≥2** | 11 (0.0) | 15 (0.5) | 6 (1.0) | 7 (4.6) | 10 (11.9) | 0 (0.0) | 2 (0.4) | 3 (2.0) | 6 (9.1) | 7 (13.7) | 3 (2.0) | 6 (9.1) | 7 (13.7) | NA | NA | NA |
| **OCS-defined exacerbations, n (%)** | | | | | | | | | | | | | | | | |
| **0** | 28,361 (98.4) | 2,994 (91.8) | 487 (83.5) | 118 (77.1) | 43 (51.2) | 1,881 (96.6) | 439 (88.0) | 115 (77.7) | 50 (75.8) | 25 (49.0) | 115 (77.7) | 50 (75.8) | 25 (49.0) | NA | NA | NA |
| **1** | 399 (1.4) | 220 (6.7) | 70 (12.0) | 20 (13.1) | 20 (23.8) | 57 (2.9) | 47 (9.4) | 22 (14.9) | 9 (13.6) | 9 (17.6) | 22 (14.9) | 9 (13.6) | 9 (17.6) | NA | NA | NA |
| **≥2** | 50 (0.2) | 49 (1.5) | 26 (4.5) | 15 (9.8) | 21 (25.0) | 9 (0.5) | 13 (2.6) | 11 (7.4) | 7 (10.6) | 17 (33.3) | 11 (7.4) | 7 (10.6) | 17 (33.3) | NA | NA | NA |

ED, emergency department; NA, not applicable; OCS, oral corticosteroid.

Asthma exacerbations were defined as any exacerbation with asthma in the primary position for exacerbations that resulted in hospitalization or an ED visit. OCS-defined exacerbations were defined as OCS prescription (England population) or any OCS prescription equivalent to prednisone 20 mg/day for 3–28 days (US population) recorded within 1 week prior to or 1 week following an asthma diagnosis code. In the England population, data for the severe refractory eosinophilic asthma group are not reported owing to the small sample size.

**Table S4.** HCRU, by exacerbation history, in the 30 days following the first severe asthma exacerbation

|  | **Treated asthma** | | | | | | **Severe asthma** | | | | | | **Severe refractory asthma** | | **Severe refractory eosinophilic asthma** | |
| --- | --- | --- | --- | --- | --- | --- | --- | --- | --- | --- | --- | --- | --- | --- | --- | --- |
|  | **Exacerbation history** | | | | | | | | | | | | | | | |
|  | **None** | | **1** | | **≥2** | | **None** | | **1** | | **≥2** | | **≥2** | | **≥2** | |
|  | **All-cause**  **HCRU** | **Asthma-related**  **HCRU** | **All-cause**  **HCRU** | **Asthma-related**  **HCRU** | **All-cause**  **HCRU** | **Asthma-related**  **HCRU** | **All-cause**  **HCRU** | **Asthma-related**  **HCRU** | **All-cause**  **HCRU** | **Asthma-related**  **HCRU** | **All-cause**  **HCRU** | **Asthma-related**  **HCRU** | **All-cause**  **HCRU** | **Asthma-related**  **HCRU** | **All-cause**  **HCRU** | **Asthma-related**  **HCRU** |
| **US population** | | | | | | | | | | | | |  |  |  |  |
| **Office visit, n (%)** | | | | | | | | | | | | | | | | |
| **0** | 1,976 (43.4) | 3,318 (72.8) | 1,354 (43.7) | 2,203 (71.1) | 858 (44.0) | 1,328 (68.1) | 305 (36.7) | 574 (69.1) | 267 (37.0) | 483 (67.0) | 253 (37.0) | 439 (64.2) | 253 (37.0) | 439 (64.2) | 6 (18.8) | 13 (40.6) |
| **1** | 1,394 (30.6) | 1,003 (22.0) | 950 (30.7) | 720 (23.2) | 571 (29.3) | 480 (24.6) | 272 (32.7) | 204 (24.5) | 218 (30.2) | 179 (24.8) | 224 (32.7) | 182 (26.6) | 224 (32.7) | 182 (26.6) | 16 (50.0) | 13 (40.6) |
| **≥2** | 1,185 (26.0) | 234 (5.1) | 794 (25.6) | 175 (5.6) | 522 (26.8) | 143 (7.3) | 254 (30.6) | 53 (6.4) | 236 (32.7) | 59 (8.2) | 207 (30.3) | 63 (9.2) | 207 (30.3) | 63 (9.2) | 10 (31.3) | 6 (18.8) |
| **Outpatient hospital visit, n (%)** | | | | | | | | | | | | | | | | |
| **0** | 3,956 (86.8) | 4,379 (96.1) | 2,674 (86.3) | 2,936 (94.8) | 1,601 (82.1) | 1,787 (91.6) | 700 (84.2) | 790 (95.1) | 590 (81.8) | 663 (92.0) | 530 (77.5) | 591 (86.4) | 530 (77.5) | 591 (86.4) | 24 (75.0) | 26 (81.3) |
| **1** | 435 (9.5) | 157 (3.4) | 317 (10.2) | 139 (4.5) | 257 (13.2) | 138 (7.1) | 92 (11.1) | 33 (4.0) | 88 (12.2) | 45 (6.2) | 109 (15.9) | 79 (11.5) | 109 (15.9) | 79 (11.5) | 3 (9.4) | 6 (18.8) |
| **≥2** | 164 (3.6) | 19 (0.4) | 107 (3.5) | 23 (0.7) | 93 (4.8) | 26 (1.3) | 39 (4.7) | 8 (1.0) | 43 (6.0) | 13 (1.8) | 45 (6.6) | 14 (2.0) | 45 (6.6) | 14 (2.0) | 5 (15.6) | 0 (0.0) |
| **ED visit, n (%)** | | | | | | | | | | | | | | | | |
| **0** | 4,263 (93.6) | 4,481 (98.4) | 2,891 (93.3) | 3,041 (98.2) | 1,788 (91.6) | 1,884 (96.6) | 769 (92.5) | 816 (98.2) | 647 (89.7) | 699 (96.9) | 619 (90.5) | 653 (95.5) | 619 (90.5) | 653 (95.5) | 30 (93.8) | 30 (93.8) |
| **1** | 244 (5.4) | 70 (1.5) | 165 (5.3) | 54 (1.7) | 139 (7.1) | 59 (3.0) | 49 (5.9) | 14 (1.7) | 60 (8.3) | 22 (3.1) | 54 (7.9) | 27 (3.9) | 54 (7.9) | 27 (3.9) | 1 (3.1) | 2 (6.3) |
| **≥2** | 48 (1.1) | 4 (0.1) | 42 (1.4) | 3 (0.1) | 24 (1.2) | 8 (0.4) | 13 (1.6) | 1 (0.1) | 14 (1.9) | 0 (0.0) | 11 (1.6) | 4 (0.6) | 11 (1.6) | 4 (0.6) | 1 (3.1) | 0 (0.0) |
| **Inpatient hospital visit, n (%)** | | | | | | | | | | | | | | | | |
| **0** | 4,367 (95.9) | 4,448 (97.7) | 2,988 (96.4) | 3,034 (97.9) | 1,835 (94.1) | 1,861 (95.4) | 800 (96.3) | 807 (97.1) | 699 (96.9) | 707 (98.1) | 632 (92.4) | 643 (94.0) | 632 (92.4) | 643 (94.0) | 30 (93.8) | 31 (96.9) |
| **1** | 174 (3.8) | 107 (2.3) | 105 (3.4) | 62 (2.0) | 108 (5.5) | 87 (4.5) | 30 (3.6) | 24 (2.9) | 21 (2.9) | 13 (1.8) | 47 (6.9) | 38 (5.6) | 47 (6.9) | 38 (5.6) | 2 (6.3) | 1 (3.1) |
| **≥2** | 14 (0.3) | 0 (0.0) | 5 (0.2) | 2 (0.1) | 8 (0.4) | 3 (0.2) | 1 (0.1) | 0 (0.0) | 1 (0.1) | 1 (0.1) | 5 (0.7) | 3 (0.4) | 5 (0.7) | 3 (0.4) | 0 (0.0) | 0 (0.0) |
| **Prescription medicine dispensed, n (%)** | | | | | | | | | | | | | | | | |
| **0** | 1,773 (38.9) | 2,561 (56.2) | 1,189 (38.4) | 1,647 (53.2) | 706 (36.2) | 929 (47.6) | 247 (29.7) | 374 (45.0) | 199 (27.6) | 297 (41.2) | 187 (27.3) | 247 (36.1) | 187 (27.3) | 247 (36.1) | 6 (18.8) | 9 (28.1) |
| **1** | 1,108 (24.3) | 1,167 (25.6) | 715 (23.1) | 776 (25.0) | 412 (21.1) | 491 (25.2) | 199 (23.9) | 240 (28.9) | 160 (22.2) | 188 (26.1) | 131 (19.2) | 181 (26.5) | 131 (19.2) | 181 (26.5) | 4 (12.5) | 6 (18.8) |
| **2** | 734 (16.1) | 552 (12.1) | 518 (16.7) | 418 (13.5) | 340 (17.4) | 328 (16.8) | 141 (17.0) | 125 (15.0) | 138 (19.1) | 139 (19.3) | 130 (19.0) | 142 (20.8) | 130 (19.0) | 142 (20.8) | 6 (18.8) | 9 (28.1) |
| **3** | 452 (9.9) | 202 (4.4) | 328 (10.6) | 193 (6.2) | 236 (12.1) | 142 (7.3) | 106 (12.8) | 61 (7.3) | 101 (14.0) | 73 (10.1) | 95 (13.9) | 72 (10.5) | 95 (13.9) | 72 (10.5) | 5 (15.6) | 3 (9.4) |
| **≥4** | 488 (10.7) | 73 (1.6) | 348 (11.2) | 64 (2.1) | 257 (13.2) | 61 (3.1) | 138 (16.6) | 31 (3.7) | 123 (17.1) | 24 (3.3) | 141 (20.6) | 42 (6.1) | 141 (20.6) | 42 (6.1) | 11 (34.4) | 5 (15.6) |
| **England population** | | | | | | | | | | | | |  |  |  |  |
| **Office visit, n (%)** | | | | | | | | | | | | | | | | |
| **0** | 194 (29.2) | 560 (84.3) | 96 (26.7) | 285 (79.2) | 54 (20.6) | 218 (83.2) | 23 (24.2) | 79 (83.2) | 23 (27.1) | 64 (75.3) | 21 (18.6) | 94 (83.2) | 21 (18.6) | 94 (83.2) | NA | NA |
| **1** | 196 (29.5) | 93 (14.0) | 105 (29.2) | 65 (18.1) | 71 (27.1) | 36 (13.7) | 24 (25.3) | 14 (14.7) | 20 (23.5) | 19 (22.4) | 21 (18.6) | 17 (15.0) | 21 (18.6) | 17 (15.0) | NA | NA |
| **≥2** | 274 (41.3) | 11 (1.7) | 159 (44.2) | 10 (2.8) | 137 (52.3) | 8 (3.1) | 48 (50.5) | 2 (2.1) | 42 (49.4) | 2 (2.4) | 71 (62.8) | 2 (1.8) | 71 (62.8) | 2 (1.8) | NA | NA |
| **ED visit, n (%)** | | | | | | | | | | | | | | | | |
| **0** | 650 (97.9) | 650 (97.9) | 354 (98.3) | 354 (98.3) | 251 (95.8) | 251 (95.8) | 94 (98.9) | 94 (98.9) | 83 (97.6) | 83 (97.6) | 105 (92.9) | 105 (92.9) | 105 (92.9) | 105 (92.9) | NA | NA |
| **1** | 14 (2.1) | 14 (2.1) | 6 (1.7) | 6 (1.7) | 9 (3.4) | 9 (3.4) | 1 (1.1) | 1 (1.1) | 2 (2.4) | 2 (2.4) | 6 (5.3) | 6 (5.3) | 6 (5.3) | 6 (5.3) | NA | NA |
| **≥2** | 0 (0.0) | 0 (0.0) | 0 (0.0) | 0 (0.0) | 2 (0.8) | 2 (0.8) | 0 (0.0) | 0 (0.0) | 0 (0.0) | 0 (0.0) | 2 (1.8) | 2 (1.8) | 2 (1.8) | 2 (1.8) | NA | NA |
| **Hospitalization, n (%)** | | | | | | | | | | | | | | | | |
| **0** | 632 (95.2) | 647 (97.4) | 342 (95.0) | 349 (96.9) | 226 (86.3) | 234 (89.3) | 87 (91.6) | 92 (96.8) | 81 (95.3) | 83 (97.6) | 90 (79.6) | 94 (83.2) | 90 (79.6) | 94 (83.2) | NA | NA |
| **1** | 26 (3.9) | 14 (2.1) | 14 (3.9) | 10 (2.8) | 29 (11.1) | 22 (8.4) | 4 (4.2) | 1 (1.1) | 3 (3.5) | 2 (2.4) | 18 (15.9) | 15 (13.3) | 18 (15.9) | 15 (13.3) | NA | NA |
| **≥2** | 6 (0.9) | 3 (0.5) | 4 (1.1) | 1 (0.3) | 7 (2.7) | 6 (2.3) | 4 (4.2) | 2 (2.1) | 1 (1.2) | 0 (0.0) | 5 (4.4) | 4 (3.5) | 5 (4.4) | 4 (3.5) | NA | NA |
| **Prescription medicine dispensed, n (%)** | | | | | | | | | | | | | | | | |
| **0** | 295 (44.4) | 370 (55.7) | 151 (41.9) | 175 (48.6) | 80 (30.5) | 98 (37.4) | 28 (29.5) | 39 (41.1) | 18 (21.2) | 26 (30.6) | 23 (20.4) | 31 (27.4) | 23 (20.4) | 31 (27.4) | NA | NA |
| **1** | 225 (33.9) | 214 (32.2) | 117 (32.5) | 133 (36.9) | 100 (38.2) | 111 (42.4) | 34 (35.8) | 36 (37.9) | 37 (43.5) | 39 (45.9) | 45 (39.8) | 52 (46.0) | 45 (39.8) | 52 (46.0) | NA | NA |
| **2** | 102 (15.4) | 63 (9.5) | 57 (15.8) | 38 (10.6) | 49 (18.7) | 34 (13.0) | 21 (22.1) | 15 (15.8) | 21 (24.7) | 15 (17.6) | 30 (26.5) | 20 (17.7) | 30 (26.5) | 20 (17.7) | NA | NA |
| **3** | 32 (4.8) | 16 (2.4) | 23 (6.4) | 11 (3.1) | 18 (6.9) | 17 (6.5) | 9 (9.5) | 5 (5.3) | 7 (8.2) | 4 (4.7) | 6 (5.3) | 8 (7.1) | 6 (5.3) | 8 (7.1) | NA | NA |
| **≥4** | 10 (1.5) | 1 (0.2) | 12 (3.3) | 3 (0.8) | 15 (5.7) | 2 (0.8) | 3 (3.2) | 0 (0.0) | 2 (2.4) | 1 (1.2) | 9 (8.0) | 2 (1.8) | 9 (8.0) | 2 (1.8) | NA | NA |

ED, emergency department; HCRU, healthcare resource utilization; NA, not applicable.

In the England population, data for the severe refractory eosinophilic asthma group are not reported owing to the small sample size.

**Figure S1.** Treatments prescribed within 30 days following the first hospital discharge after an asthma exacerbation in A) the US and b) England


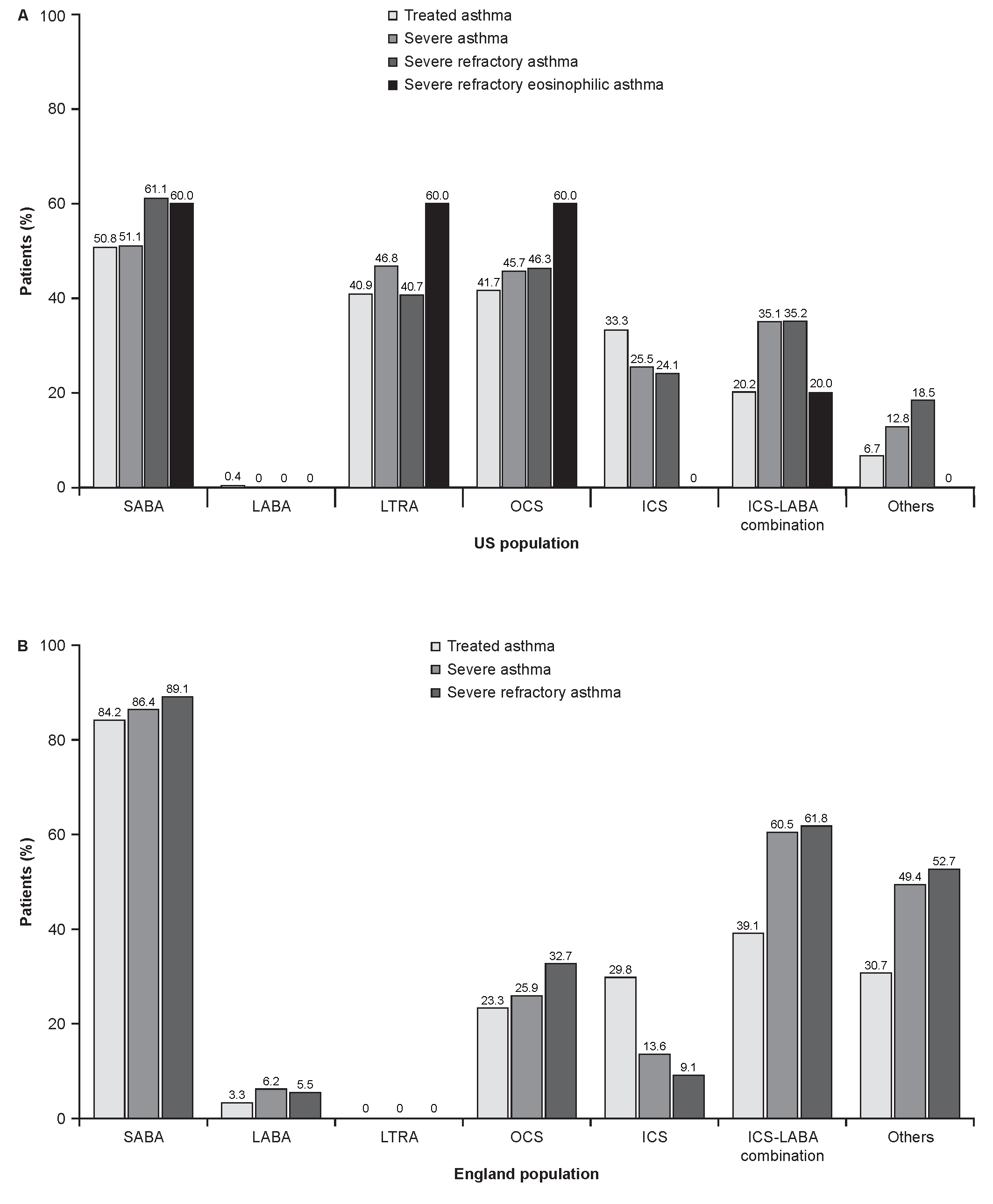


ICS, inhaled corticosteroid; LABA, long-acting beta-agonist; LTRA, leukotriene receptor antagonist; OCS, oral corticosteroid; SABA, short-acting beta-agonist.

US treated asthma group: n=151,549; US severe asthma group: n=18,086; US severe refractory asthma group: n=2,099; US severe refractory eosinophilic asthma group: n=109; England treated asthma group: n=32,893; England severe asthma group: n=2,711; England severe refractory asthma group: n=265. In the England population, data for the severe refractory eosinophilic asthma group are not reported owing to the small sample size.
